# Supplementary material for: Excellence in Communication and Emergency Leadership (ExCEL): Pediatric Primary and Secondary Survey in Trauma Workshop for Residents
Source: MedEdPORTAL. 2021 Jan 22;17:11079. doi: 10.15766/mep_2374-8265.11079 (PMC7821439; doi:10.15766/mep_2374-8265.11079)
Supplement: Supplementary file 1 — ExCEL Trauma Survey Workshop Survey.docxTrauma Survey Demonstration.docxRole-Play Prebrief.docxNormal Trauma Survey.docxInjured Patient Trauma Survey.docx [file mep_2374-8265.11079-s001.zip › _Educational_Summary_Report_11079.pdf]

MedEdPORTAL® The AAMC Journal of Teaching and Learning Resources

Original Publication Open Access

Excellence in Communication and Emergency Leadership (ExCEL): Pediatric Primary and Secondary Survey in Trauma Workshop for Residents

Mariann Nocera Kelley, MD*, Laura Mercurio, MD, Hoi See Tsao, MD, Vanessa Toomey, MD, MBA, Marie Carillo, MD, Linda Brown, MD, MSCE, Robyn Wing, MD, MPH

*Corresponding author: mkelley@connecticutchildrens.org

Abstract

Introduction: Unintentional traumatic injury remains the leading cause of pediatric death in the United States. There is wide variation in the assessment and management of pediatric trauma patients in emergency departments. Resident education on trauma evaluation and management is lacking. This workshop focused on developing resident familiarity with the primary and secondary trauma survey in pediatric patients. **Methods:** This hands-on workshop utilized patient-actors and low-fidelity simulators to instruct learners on the initial assessment of trauma patients during the primary and secondary trauma surveys. It was designed for residents across all levels of training who care for pediatric trauma patients (including pediatrics, medicine-pediatrics, emergency medicine, and family medicine) and adapted for different session durations and learner group sizes. **Results:** Eighteen residents participated in this workshop at two separate institutions. Participants strongly agreed that the workshop was relevant and effective in teaching the initial primary and secondary trauma survey assessment of pediatric trauma patients. Residents also reported high levels of confidence in performing a primary and secondary trauma survey after participation in the workshop. **Discussion:** This workshop provided residents with instruction and practice in performing the primary and secondary trauma survey for injured pediatric patients. Additional instruction is needed on assigning Glasgow Coma Scale and AVPU (alert, voice, pain, unresponsive) scores to injured patients. The structure and time line of this curriculum can be adapted to the needs of an individual institution's program and the number of workshop participants.

Keywords
Pediatric Residents, Pediatrics, Emergency, Pediatric Emergency Medicine, Emergency Treatment, Pediatric Trauma, Primary Trauma Survey, Secondary Trauma Survey

Educational Objectives

By the end of this activity, learners will be able to:

1. Recall steps included in the primary and secondary surveys of trauma patients.
2. Assign a Glasgow Coma Scale score to trauma patients with varying degrees of altered mental status.
3. Demonstrate the ability to perform primary and secondary surveys.
4. Develop plans for management of injured patients.

Citation:
Kelley MN, Mercurio L, Tsao HS, et al. Excellence in Communication and Emergency Leadership (ExCEL): pediatric primary and secondary survey in trauma workshop for residents. *MedEdPORTAL*. 2021;17:11079. https://doi.org/10.15766/mep_2374-8265.11079

Introduction

Unintentional traumatic injury remains the leading cause of pediatric death in the United States.[1] According to the annual pediatric report from the National Trauma Data Bank, 141,051 pediatric trauma cases were reported in 2016, with 3,461 associated deaths.[2] In the majority of cases, these children received care outside of a pediatric trauma center.[2,3] As a result, there is a growing emphasis on pediatric readiness, as well as acknowledgment of the increased morbidity and mortality associated with underprepared institutions.[3-5]

Pediatric trauma care varies widely among emergency departments, and prior literature has identified multiple deficiencies in pediatric trauma management.[6-8] Residency program directors report insufficient education in trauma assessment.[9] The trauma assessments performed by pediatric and emergency medicine residents are often incomplete.[10] This may be due to limited exposure to trauma resuscitations in the

Copyright © 2021 Kelley et al. This is an open-access publication distributed under the terms of the Creative Commons Attribution license.

clinical setting.[9] Critical examination of trauma care has also found that poor team leadership, poor team organization, and deviation from Advanced Trauma Life Support (ATLS) protocols result in inefficient trauma assessments.[11-15] Rapid assessments are associated with improved clinical outcomes in trauma resuscitations.[13-15] These factors highlight a need for additional training for residents on trauma assessment.[9,10]

Simulation is a well-established method of exposing trainees to new skills or rare clinical scenarios. Residents report that simulation improves their comfort with caring for critically ill children.[14] Emergency departments throughout the United States have demonstrated enhanced trauma resuscitation skills,[6-8] as well as improved team leadership,[11,16,17] through simulation training. Simulation-based trauma curricula have also been shown to improve resident comfort with trauma resuscitation skills,[18,19] as well as to improve clinical performance, when compared to traditional didactic training.[20]

This workshop is part of a series from our larger curriculum called Excellence in Communication and Emergency Leadership, or ExCEL.[21] The ExCEL curriculum was developed with a goal of replacing standard resident morning report didactics once a month with simulation-based training and core resuscitation skills sessions. The ExCEL curriculum aims to augment and support the clinical skills obtained through training while helping residents gain confidence, sharpen and maintain critical technical skills, and improve leadership and communication proficiency. The monthly small-group workshops incorporate case-based learning, active commitment exercises, and hands-on practice of technical skills. This Pediatric Primary and Secondary Survey in Trauma Workshop can be used independently or in conjunction with other sessions from the ExCEL curriculum.

Given the importance of prompt recognition and management of injuries in pediatric trauma patients,[22] we developed a novel curriculum to review the critical steps for residents assessing these patients. While there are many trauma simulations published in *MedEdPORTAL*,[23-27] there are no published curricula designed to instruct residents on performing the initial trauma evaluation of pediatric patients. The primary goal of this workshop was to improve both resident understanding of the primary and secondary trauma evaluation and resident comfort in performing this assessment. The workshop utilized social learning theory, experiential learning theory, and reflective learning to allow for both active and passive learning experiences.[28] The session was replicated at regular intervals throughout the academic year to reinforce knowledge and skills, increase comfort with trauma evaluations, and ideally translate into improved patient care.

Methods

Target Audience

The target audience for our curriculum included PGY 1-PGY 5 pediatric, medicine-pediatric, triple board (pediatrics, psychiatry, and child and adolescent psychiatry), family medicine, and emergency medicine residents. Other learners, including rotating third- and fourth-year medical students, frequently attended these educational sessions but were outside of our target population. We ran this workshop at two different institutions, adapting the workshop to the needs and structure of the morning report at each institution.

Instructor/Facilitator

This workshop was run with a combination of instructors acting as patient-actors and/or manikins. Instructors had knowledge of ATLS and training in medical education. Though manikins were used to practice the examination, formal training in simulation for the instructors was not necessary. Instructors also had experience with the resources and systems in place for trauma evaluation and management at their specific institution. They included pediatric emergency medicine attendings, fellows, and pediatric chief residents. Pediatric surgery attendings and fellows would also be qualified instructors. This session required at least one instructor to facilitate the session and provide feedback on trauma survey performance. If additional instructors were available to act as patient-actors, they assisted in case facilitation and performance feedback. When additional instructors were not available to act as patient-actors, manikins were used, with the instructor providing the details of the examination during the trauma surveys. When an abundance of instructors was available, multiple parallel groups were run simultaneously, which allowed us to accommodate more learners per session.

Setting

The workshop took place in an empty patient room or conference room large enough to fit a stretcher or table sufficient to support an adult patient-actor or manikin.

Time Line

Given the hands-on nature of this workshop, the time necessary for completion depended upon the number of learners participating. At one institution, a 60-minute time frame was necessary due to educational time restraints in our learners' clinical day; our sessions took place during typical morning report hours. This time frame allowed for up to 15 learners. However, this workshop time line could be extended to accommodate

Copyright © 2021 Kelley et al. This is an open-access publication distributed under the terms of the Creative Commons Attribution license.

additional learners. At a second institution with a smaller number of learners and instructors, the workshop was completed in a 30-minute time frame. Participants at each institution were divided into groups of three to four learners per patient-actor or manikin to provide for maximum hands-on learning. For more detail, see Table 1.

Preparation

Evaluation: Instructors reviewed materials and printed out Appendix A (ExCEL Trauma Survey Workshop Survey) for each participant. Alternatively, instructors could transform the survey into an electronic format for ease of data collection.

Equipment: In advance of the workshop, instructors identified and secured the necessary equipment for this workshop, including the following:

- Low-fidelity manikin (if no patient-actors): The manikin needed to allow for a simple physical exam. We used a Gaumard Code Blue III Pediatric manikin. However, other similar systems would also be adequate.
- Stethoscopes.
- Otoscopes/specula.
- C-collars (appropriately sized for patient-actor or manikin).
- Stretchers or tables for patient-actor(s) or manikin(s).

On the day of the workshop, instructors arrived early for setup of tables and manikins, if used.

Content

In the large-group introduction, the instructor(s) demonstrated the primary and secondary trauma survey with both an ideal example and a nonideal example of a trauma evaluation, based on the ATLS curriculum from the American College of Surgeons.[22] (See Appendix B.)

The learners next split into smaller groups of three to four participants and one patient-actor or manikin. First, the facilitator provided a prebrief to participants to explain the role of the patient-actor or manikin in the patient assessment (Appendix C). Each group member then performed a normal primary and secondary trauma survey (Appendix D) to allow every individual the opportunity to practice a normal trauma survey without injuries. Three additional trauma cases were run in each group, with each case led by a different group member who self-assigned as leader (Appendix E). This allowed participants to demonstrate the management of injured trauma patients. When a patient-actor was used, she or he verbally provided the physical exam findings for each case; when a manikin was used, the facilitator provided the physical exam findings for each case. Individuals not actively participating observed the other participants, provided help to those performing the trauma surveys, and offered feedback at the end. Facilitators gave feedback to the participants at the conclusion of each case. A short debriefing was conducted using the rapid-cycle deliberate practice model.[29]

ExCEL Curriculum

As previously mentioned, this workshop was part of a series from our larger ExCEL curriculum.[21] As an unordered series, this workshop could be given at any time in the curriculum. The workshops were planned 6 months in advance but could be adjusted if facilitators identified a clinical concern on a relevant topic that would benefit from an educational intervention. The Pediatric Primary and Secondary Survey in Trauma Workshop for Residents was typically run twice a year.

Evaluation

Residents were asked to complete the ExCEL Trauma Survey Workshop Survey (Appendix A) as an evaluation of the session and to obtain feedback to ensure our goals and objectives were met, as perceived by the learners. This form also solicited feedback about participants' suggestions for improvements of the session. For ease of use and to maximize response rates, we converted the survey into an electronic form easily accessible with a QR code.

Results

This workshop was conducted at two separate institutions with a total of 18 resident participants. The workshop's length and format were adapted to the needs and structure of the morning report at each institution. At the first institution, the workshop included 15 resident participants and four instructors and ran

Table 1. Suggested Time Line for Pediatric Primary and Secondary Survey Workshop

Time Line			
60-Minute Workshop	30-Minute Workshop	Item	Activity
5 minutes	5 minutes	Introduction	Introduction/demonstration of primary and secondary trauma surveys
25 minutes	10 minutes	Normal trauma scenario	Individual practice performing a trauma evaluation
25 minutes	10 minutes	Mass trauma scenarios	In groups, evaluations of injured patients
5 minutes	5 minutes	Wrap-up and workshop evaluation	Large-group discussion/completion of evaluation

Copyright © 2021 Kelley et al. This is an open-access publication distributed under the terms of the Creative Commons Attribution license.

over 60 minutes. At the second institution, the workshop included three resident participants and one instructor and ran over 30 minutes.

Of these participants, nine (50%) were interns (PGY 1), and nine (50%) were residents (PGY 2 and above). Based on our evaluation form, we received very positive feedback about learner satisfaction and self-efficacy to perform the tasks in the educational objectives (Tables 2 and 3).

Participants stated that they found the ability to practice hands-on trauma scenarios most helpful. They also felt that the session could be improved upon with more time spent reviewing the Glasgow Coma Scale (GCS).

Discussion

Unintentional trauma is the leading cause of pediatric death in the United States.[1] In a pediatric emergency department, residents often perform the initial trauma evaluation, although there are variations in training and common deficiencies in their performance.[6-8] The Pediatric Primary and Secondary Survey in Trauma Workshop is an effective educational intervention for residents caring for injured children. The skills reinforced in this workshop, including how to perform a primary and secondary pediatric trauma survey and recognize abnormalities in these assessments, are vital for any resident managing an injured child.

Residents charged with performing an initial trauma evaluation often do not get sufficient education and subsequently have been shown to perform incomplete assessments, most likely due to limited trauma exposure in the clinical setting.[9,10] After participation in our workshop, the majority of residents involved felt confident in their ability to perform a primary and secondary survey on a trauma patient. In addition, more than half reported confidence in their ability to develop plans for the management of injured pediatric patients. Instructors anecdotally observed a positive trend in residents stepping up to perform trauma surveys in the trauma bay and performing efficient yet complete primary and secondary surveys.

Table 2. Participant Feedback on Primary and Secondary Survey in Trauma Workshop ($n = 18$)

Statement	Mean Score[a]
This workshop was relevant to my work.	4.8
This workshop was effective in teaching me the steps of the primary and secondary surveys for trauma patients.	4.9
This workshop was effective in teaching me how to assign a Glasgow Coma Scale to trauma patients.	3.8

[a]Rated on a 5-point Likert scale (1 = *strongly disagree*, 5 = *strongly agree*).

Table 3. Participant Feedback on Self-Confidence Related to Learning Objectives ($n = 18$)

Question	Median Score[a]	% of Respondents Answering 4 or 5[a]
After participating in this session, how confident are you in your ability to:		
Perform a primary and secondary survey on a trauma patient?	4.0	83
Assign a Glasgow Coma Scale to a trauma patient?	3.5	50
Develop plans for management of injury patients?	4.0	61

[a]Rated on a 5-point Likert scale (1 = *very not confident*, 5 = *very confident*).

Only half of the participants felt confident in their ability to assign a GCS score to a patient. As a result, we have adapted our instructional techniques to include more focus on the assignment of a GCS score to our simulated patients. Directing appropriate attention to both performance of the primary and secondary surveys and assignment of a GCS score proved difficult in the shorter version of the workshop due to time constraints. Given prior literature highlighting limited resident education in primary and secondary surveys, this particular objective was emphasized in the shorter workshop. In future iterations, the objective of GCS assignment may be removed from the shorter workshop or sent out for review in a prelearning assignment. Future iterations will also include AVPU (alert, voice, pain, unresponsive), which could be incorporated into the flipped classroom model as well.

Our experience at multiple institutions revealed that the workshop's format and group sizes were determined by the number of available instructors. Live patient-actors (instructors) were used as patients in some iterations, allowing for more realistic feedback on the physical exam compared to a simulation manikin. The shorter workshop did not allow for multiple scenarios to be run, and therefore, the participants did not get the benefit of repetition. Furthermore, with a manikin serving as the patient, the resident participants had to rely solely on the facilitator to provide the exam findings, which limited the residents' ability to call out their findings as would be typical in a real trauma situation. The ideal instructor-to-learner ratio is therefore two instructors for each group of three to five residents; this allows one instructor to act as the live patient-actor, and the second instructor to facilitate the trauma surveys and observe within the small-group setting. With more instructors, more groups can practice hands-on skills simultaneously, allowing for more case scenarios to be run within the given time frame.

Future iterations of the workshop can be modified to include a flipped classroom model. Introducing the fundamentals of the primary and secondary trauma surveys prior to the session would allow more time for hands-on practice for the participants.

Copyright © 2021 Kelley et al. This is an open-access publication distributed under the terms of the Creative Commons Attribution license.

It would also allow for the inclusion of a broader range of topics, including GCS and AVPU. Finally, adding more diverse patient cases with more patient ages would allow for broader instruction on pediatric-specific challenges in trauma evaluations, including pediatric-specific developmental considerations, physiology, and patterns of injury.

The main limitations of our workshop were the participants' varied levels of trainings and variation in their prior experience with trauma surveys. Emergency medicine residents reported more exposure to trauma than pediatric residents but may have had less familiarity and comfort with caring for injured children. The session length also provided a slightly different experience for the participants at different institutions, although it did demonstrate that the workshop can be adapted to fit the needs and resources of individual institutions. Finally, this course was not designed to replace an ATLS certification course and did not include education about cervical spine clearance. Pediatric, combined internal medicine and pediatrics, and family medicine residents may not be required to get ATLS certification, and this course was not designed to provide comprehensive trauma assessment and management education. It can, however, serve as a supplement to any formalized trauma training that residents may receive or as an introduction to trauma management for residents who are not certified in ATLS. Another limitation is that there was no direct assessment of our stated learning objectives. The course allowed participants the opportunity to recall steps, perform them in a role-play environment, and develop a management plan for patient cases. However, there was no formal evaluation since the rapid-cycle deliberate practice debriefing discussion gave participants real-time formative feedback.

This workshop was designed as part of the larger ExCEL curriculum and therefore can be implemented as a component of a larger morning report curriculum, or it can stand on its own as an independent educational tool. The workshop augments ATLS training and pediatric emergency medicine education while providing an opportunity for residents to receive hands-on training in the management of injured pediatric patients. Based on resident feedback, future iterations of this workshop will incorporate more discussion on assigning a GCS or AVPU score.

Appendices

A. ExCEL Trauma Survey Workshop Survey.docx

B. Trauma Survey Demonstration.docx

C. Role-Play Prebrief.docx

D. Normal Trauma Survey.docx

E. Injured Patient Trauma Survey.docx

All appendices are peer reviewed as integral parts of the Original Publication.

Mariann Nocera Kelley, MD: Assistant Professor, Departments of Pediatrics and Emergency Medicine/Traumatology, Division of Pediatric Emergency Medicine, University of Connecticut School of Medicine and Connecticut Children's; Director of Simulation Education, University of Connecticut School of Medicine

Laura Mercurio, MD: Fellow, Departments of Emergency Medicine and Pediatrics, Division of Pediatric Emergency Medicine, Warren Alpert Medical School of Brown University and Rhode Island Hospital/Hasbro Children's Hospital

Hoi See Tsao, MD: Fellow, Departments of Emergency Medicine and Pediatrics, Division of Pediatric Emergency Medicine, Warren Alpert Medical School of Brown University and Rhode Island Hospital/Hasbro Children's Hospital

Vanessa Toomey, MD, MBA: Clinical Fellow, Department of Anesthesiology, Critical Care, and Pain Medicine, Harvard Medical School and Boston Children's Hospital

Marie Carillo, MD: Fellow, Department of Cardiology, Children's National Hospital

Linda Brown, MD, MSCE: Associate Professor, Departments of Emergency Medicine and Pediatrics, Division of Pediatric Emergency Medicine, Warren Alpert Medical School of Brown University and Rhode Island Hospital/Hasbro Children's Hospital; Vice Chair of Pediatric Emergency Medicine, Hasbro Children's Hospital; Director, Lifespan Medical Simulation Center

Robyn Wing, MD, MPH: Assistant Professor, Departments of Emergency Medicine and Pediatrics, Division of Pediatric Emergency Medicine, Warren Alpert Medical School of Brown University and Rhode Island Hospital/Hasbro Children's Hospital; Director of Pediatric Simulation, Lifespan Medical Simulation Center

Acknowledgments
Dr. Linda Brown and Dr. Robyn Wing are co-senior authors.

Disclosures
None to report.

Funding/Support
None to report.

Ethical Approval
Reported as not applicable.

Copyright © 2021 Kelley et al. This is an open-access publication distributed under the terms of the Creative Commons Attribution license.

References

1. Kochanek KD, Murphy SL, Xu JQ, Arias E. Deaths: final data for 2017. *Natl Vital Stat Rep.* 2019;68(9):1-76.
2. McDermott KW, Stocks C, Freeman WJ. *Overview of Pediatric Emergency Department Visits, 2015: HCUP Statistical Brief 242. Agency for Healthcare Research and Quality*; 2018. www.hcupus.ahrq.gov/reports/statbriefs/sb242-Pediatric-ED-Visits-2015.pdf
3. Petrosyan M, Guner YS, Emami CN, Ford HR. Disparities in the delivery of pediatric trauma care. *J Trauma Inj Infect Crit Care*. 2009;67(2):S114-S119. https://doi.org/10.1097/TA.0b013e3181ad3251
4. Ames SG, Davis BS, Marin JR, et al. Emergency department pediatric readiness and mortality in critically ill children. *Pediatrics*. 2019;144(3):e20190568. https://doi.org/10.1542/peds.2019-0568
5. Remick K, Gaines B, Ely M, Richards R, Fendya D, Edgerton EA. Pediatric emergency department readiness among US trauma hospitals. *J Trauma Acute Care Surg*. 2019;86(5):803-809. https://doi.org/10.1097/TA.0000000000002172
6. Hunt EA, Hohenhaus SM, Luo X, Frush KS. Simulation of pediatric trauma stabilization in 35 North Carolina emergency departments: identification of targets for performance improvement. *Pediatrics*. 2006;117(3):641-648. https://doi.org/10.1542/peds.2004-2702
7. Hunt EA, Walker AR, Shaffner DH, Miller MR, Pronovost PJ. Simulation of in-hospital pediatric medical emergencies and cardiopulmonary arrests: highlighting the importance of the first 5 minutes. *Pediatrics*. 2008;121(1):e34-e43. https://doi.org/10.1542/peds.2007-0029
8. Bayouth L, Ashley S, Brady J, et al. An in-situ simulation-based educational outreach project for pediatric trauma care in a rural trauma system. *J Pediatr Surg*. 2018;53(2):367-371. https://doi.org/10.1016/j.jpedsurg.2017.10.042
9. Trainor JL, Krug SE. The training of pediatric residents in the care of acutely ill and injured children. *Arch Pediatr Adolesc Med*. 2000;154(11):1154-1159. https://doi.org/10.1001/archpedi.154.11.1154
10. Gala PK, Osterhoudt K, Myers SR, Colella M, Donoghue A. Performance in trauma resuscitation at an urban tertiary level I pediatric trauma center. *Pediatr Emerg Care*. 2016;32(11):756-762. https://doi.org/10.1097/PEC.0000000000000942
11. Capella J, Smith S, Philp A, et al. Teamwork training improves the clinical care of trauma patients. *J Surg Educ*. 2010;67(6):439-443. https://doi.org/10.1016/j.jsurg.2010.06.006
12. Steinemann S, Berg B, Skinner A, et al. In situ, multidisciplinary, simulation-based teamwork training improves early trauma care. *J Surg Educ*. 2011;68(6):472-477. https://doi.org/10.1016/j.jsurg.2011.05.009
13. Vernon DD, Furnival RA, Hansen KW, et al. Effect of a pediatric trauma response team on emergency department treatment time and mortality of pediatric trauma victims. *Pediatrics*. 1999;103(1):20-24. https://doi.org/10.1542/peds.103.1.20
14. Driscoll PA, Vincent CA. Variation in trauma resuscitation and its effect on patient outcome. *Injury*. 1992;23(2):111-115. https://doi.org/10.1016/0020-1383(92)90044-S
15. Spanjersberg WR, Bergs EA, Mushkudiani N, Klimek M, Schipper IB. Protocol compliance and time management in blunt trauma resuscitation. *Emerg Med J*. 2009;26(1):23-27. https://doi.org/10.1136/emj.2008.058073
16. Fernandez R, Rosenman ED, Olenick J, et al. Simulation-based team leadership training improves team leadership during actual trauma resuscitations: a randomized controlled trial. *Crit Care Med*. 2020;48(1):73-82. https://doi.org/10.1097/CCM.0000000000004077
17. Gregg SC, Heffernan DS, Connolly MD, et al. Teaching leadership in trauma resuscitation: immediate feedback from a real-time, competency-based evaluation tool shows long-term improvement in resident performance. *J Trauma Acute Care Surg*. 2016;81(4):729-734. https://doi.org/10.1097/TA.0000000000001186
18. Holland JR, Latuska RF, MacKeil-White K, Ciener DA, Vukovic AA. “Sim one, do one, teach one”: a simulation-based trauma orientation for pediatric residents in the emergency department. *Pediatr Emerg Care*. Published online 2020. https://doi.org/10.1097/PEC.0000000000002003
19. Mikrogianakis A, Osmond MH, Nuth JE, Shephard A, Gaboury I, Jabbour M. Evaluation of a multidisciplinary pediatric mock trauma code educational initiative: a pilot study. *J Trauma Inj Infect Crit Care*. 2008;64(3):761-767. https://doi.org/10.1097/TA.0b013e3180341ff8
20. Knudson MM, Khaw L, Bullard MK, et al. Trauma training in simulation: translating skills from SIM time to real time. *J Trauma Inj Infect Crit Care*. 2008;64(2):255-264. https://doi.org/10.1097/TA.0b013e31816275b0
21. Wing R, Tsao HS, Toomey V, et al. Excellence in Communication and Emergency Leadership (ExCEL): pediatric first 5 minutes workshop for residents. *MedEdPORTAL*. 2020;16:10980. https://doi.org/10.15766/mep_2374-8265.10980
22. ATLS Subcommittee, American College of Surgeons’ Committee on Trauma; International ATLS Working Group. Advanced Trauma Life Support (ATLS): the ninth edition. *J Trauma Acute Care Surg*. 2013;74(5):1363-1366. https://doi.org/10.1097/TA.0b013e31828b82f5
23. Szyld D, Peyre SE, Cooper ZR, Miller D, Michaud Y, Raja AS. Trauma team training: multidisciplinary training for trauma management. *MedEdPORTAL*. 2011;7:8267. https://doi.org/10.15766/mep_2374-8265.8267
24. Brown E, Duong DK. Assessment of the trauma patient. *MedEdPORTAL*. 2012;8:9261. https://doi.org/10.15766/mep_2374-8265.9261

Copyright © 2021 Kelley et al. This is an open-access publication distributed under the terms of the Creative Commons Attribution license.

25. Elisseou S, Puranam S, Nandi M. A novel, trauma-informed physical examination curriculum for first-year medical students. *MedEdPORTAL*. 2019;15:10799. https://doi.org/10.15766/mep_2374-8265.10799
26. Barlow B, Ten Eyck R. Pediatric trauma simulation case. *MedEdPORTAL*. 2010;6:8001. https://doi.org/10.15766/mep_2374-8265.8001
27. Falcone J, Erny N, Phrampus P, Forsythe R. a trauma resuscitation team leader training curriculum based on eight human patient simulations. *MedEdPORTAL*. 2014;10:9695. https://doi.org/10.15766/mep_2374-8265.9695
28. Taylor DCM, Hamdy H. Adult learning theories: implications for learning and teaching in medical education: AMEE Guide no. 83. *Med Teach*. 2013;35(11):e1561-e1572. https://doi.org/10.3109/0142159X.2013.828153
29. Perretta JS, Duval-Arnould J, Poling S, et al. Best practices and theoretical foundations for simulation instruction using rapid-cycle deliberate practice. *Simul Healthc*. 2020;15:5:356-362. https://doi.org/10.1097/SIH.0000000000000433

Received: May 11, 2020
Accepted: October 6, 2020
Published: January 22, 2021

Copyright © 2021 Kelley et al. This is an open-access publication distributed under the terms of the Creative Commons Attribution license.
