# Supplementary material for: Excellence in Communication and Emergency Leadership (ExCEL): Pediatric Primary and Secondary Survey in Trauma Workshop for Residents
Source: MedEdPORTAL. 2021 Jan 22;17:11079. doi: 10.15766/mep_2374-8265.11079 (PMC7821439; doi:10.15766/mep_2374-8265.11079)
Supplement: Supplementary file 1 — ExCEL Trauma Survey Workshop Survey.docxTrauma Survey Demonstration.docxRole-Play Prebrief.docxNormal Trauma Survey.docxInjured Patient Trauma Survey.docx [file mep_2374-8265.11079-s001.zip › A. ExCEL Trauma Survey Workshop Survey.docx]

**ExCEL Skills Session Survey – Trauma Primary and Secondary Survey**

1. I am a:

☐ Pediatric Intern ☐ Pediatric Resident ☐ EM Intern ☐ EM Resident

☐ Family Medicine Resident ☐ Medical Student

☐ Other _______________________

1. Have you participated in this ExCEL skills session in the past?

☐ Yes ☐ No ☐ Unsure

1. When was the last time you took PALS?

☐ <6 months ☐ 6 months – less than 1 year ☐ 1-2 years ☐ Unsure

**Please rate your agreement with the following statements:**

|  |  | Strongly Disagree | Disagree | Neither Agree nor Disagree | Agree | Strongly Agree |
| --- | --- | --- | --- | --- | --- | --- |
| 4. | This skills station was relevant to my work. | □ | □ | □ | □ | □ |
| 5. | This skills station was effective in teaching me the steps of the primary and secondary surveys for trauma patients. | □ | □ | □ | □ | □ |
| 6. | This skills station was effective in teaching me how to assign a Glasgow Coma Scale to trauma patients. | □ | □ | □ | □ | □ |

**After participating in this session, how confident are you in your ability to:**

|  |  | Very Not confident | Not confident | Neutral | Confident | Very Confident |
| --- | --- | --- | --- | --- | --- | --- |
| 7. | Perform a primary and secondary survey on a trauma patient? | □ | □ | □ | □ | □ |
| 8. | Assign a Glasgow Coma Scale to a trauma patient? | □ | □ | □ | □ | □ |
| 9. | Develop plans for management of injury patients? | □ | □ | □ | □ | □ |

10. What did you find most helpful about this skills session?

11. What do you think could be improved upon for this skills session in the future?

12. Other comments or suggestions:
