# Supplementary material for: Excellence in Communication and Emergency Leadership (ExCEL): Pediatric Primary and Secondary Survey in Trauma Workshop for Residents
Source: MedEdPORTAL. 2021 Jan 22;17:11079. doi: 10.15766/mep_2374-8265.11079 (PMC7821439; doi:10.15766/mep_2374-8265.11079)
Supplement: Supplementary file 1 — ExCEL Trauma Survey Workshop Survey.docxTrauma Survey Demonstration.docxRole-Play Prebrief.docxNormal Trauma Survey.docxInjured Patient Trauma Survey.docx [file mep_2374-8265.11079-s001.zip › B. Trauma Survey Demonstration.docx]

**Trauma Survey Demonstration**

Objective: Facilitators demonstrate an “ideal” and a “non-ideal” primary and secondary trauma survey to highlight those actions that contribute to a well-run trauma survey.

**Facilitator Actions**

Facilitator(s) should perform each step completely and efficiently. Sample scripting provided below.

**Patient-actor Actions**

The patient-actor can decide which portions of the physical examination (s)he does not feel comfortable allowing the facilitators or participants to perform. For example, patient-actors can decline to allow palpation of femoral pulses, evaluation for pelvic mobility or palpation of chest wall or extremities. This should be discussed with the facilitators ahead of time.

Normal Trauma Survey^1^

1. **Primary survey**:

| **Assessment** | **Facilitator Actions and *Statements*** |
| --- | --- |
| - 1. Airway | Ask patient a question. Get a clear response.  *State, “Airway intact”* |
| - 1. Breathing | Listen to bilateral lung fields.  *State, “Bilateral breath sounds”* |
| - 1. Circulation | Feel femoral pulses. (If patient-actor, the facilitator need only state that (s)he will palpate femoral pulse.)  *State, “2+ femoral pulses”.*  Obtain heart rate and blood pressure from nurse/tech.  *State, “Heart rate and blood pressure within normal limits for age”* |
| - 1. Disability | Ask patient name, place and date.  *State, “Alert and oriented to person, place and time”*  Observe for spontaneous eye opening, verbal response and motor response.  *State, “GCS 15”* (If using infant manikin, observe that patient is alert. *State,* *“Patient alert on AVPU, this correlates to GCS of 15”*) |
| - 1. Exposure | Remove all clothing. (If patient-actor, the facilitator need only state that (s)he will remove all clothing.)  *State, “No obvious injuries when patient exposed”* |

1. **Secondary survey**: Observe, palpate and, when necessary, auscultate

| - 1. Head | Inspect and palpate scalp to assess for tenderness, wounds, hematomas, or step-offs.  *State, “Atraumatic without tenderness”* |
| --- | --- |
| - 1. Face | Inspect for wounds, swelling or ecchymosis and palpate for tenderness or step-offs  *State, “No trauma, no tenderness, no bruises “* |
| - 1. Eyes | Use light to evaluate pupil size and reactivity.  *State, “Pupils equal, round and reactive to light – 4mm to 2mm bilaterally”* |
| - 1. Ears | Use otoscope bilaterally to evaluate for hemotympanum or bleeding of external auditory canal.  *State, “No hemotympanum bilaterally”* |
| - 1. Nose | Use otoscope bilaterally to evaluate for septal hematoma, bleeding or swelling.  *State, “No deformities, epistaxis or nasal septal hematoma”* |
| - 1. Mouth | Use light to evaluate for wounds, dental trauma. Ask the patient to bite down and describe if teeth align normally.  *State, “No bleeding or malocclusion. Teeth intact.”* |
| - 1. Neck | Palpate anterior neck for edema, swelling, and position of the trachea  *State, “Trachea midline”.*  Hold C-spine in place while palpating each posterior vertebral spine.  *State, “No midline C-spine tenderness or step-offs”* |
| - 1. Chest | Inspect chest wall for deformity, ecchymosis or wounds. Observe chest rise during breathing for symmetry. Palpate anterior chest wall for tenderness or crepitus. (If patient-actor, the facilitator need only state that (s)he will perform these steps).  *State, “Symmetric chest rise, no clavicular deformity or tenderness, no crepitus, no tenderness to palpation”* |
| - 1. Abdomen | Inspect abdomen for deformity, ecchymosis or wounds. Palpate in all 4 quadrants for tenderness. (If patient-actor, the facilitator need only state that (s)he will perform these steps).  *State, “No bruising, ecchymosis, seatbelt sign; no tenderness”* |
| - 1. Pelvis | Inspect for ecchymosis, deformity, asymmetry, or wounds. Palpate the pelvis for tenderness. Assess for mobility by compressing iliac crests gently. Inspect urethral meatus for blood. (If patient-actor, the facilitator need only state that (s)he will perform these steps).  *State, “Pelvis stable, no blood at the meatus, no perineal bruising or lacerations”* |
| - 1. Back | Inspect for ecchymosis, deformity, asymmetry or wounds. Palpate along spine for tenderness or step-offs.  *State, “No bruising, nontender without step-offs. Good gluteal tone”* |
| - 1. Extremities | For each extremity: Inspect for ecchymosis, deformity, or wounds. Palpate for tenderness.  *State, “No bruising, lacerations, deformities; no tenderness to upper and lower extremities.”*  For each extremity: Assess strength and sensation.  *State, “Strength 5/5 in upper and lower extremities bilaterally; sensation 5/5 in upper and lower extremities bilaterally”*  For each extremity: Assess range of motion of all joints  *State, “Full range of motion in all extremities”* |

**“Non-ideal” example** of a normal trauma survey

While examining the simulated patient (patient-actor or manikin), the facilitator:

- Speaks quietly or incomprehensibly
- Misses essential components of the trauma survey, eliminating entire portions of the exam
- Speaks slowly, performs unnecessary portions of the examination
- Becomes distracted

**“Ideal” example** of a normal trauma survey

While examining the simulated patient (patient-actor or manikin), the facilitator:

- Speaks loudly and clearly
- Completes all elements of the trauma survey, as above
- Commands control of the room, including side conversations

**Reference**:

1. Advanced trauma life support (ATLS®). *J Trauma Acute Care Surg*. 2013;74(5):1363-1366. doi:10.1097/TA.0b013e31828b82f5S
