# Supplementary material for: Excellence in Communication and Emergency Leadership (ExCEL): Pediatric Primary and Secondary Survey in Trauma Workshop for Residents
Source: MedEdPORTAL. 2021 Jan 22;17:11079. doi: 10.15766/mep_2374-8265.11079 (PMC7821439; doi:10.15766/mep_2374-8265.11079)
Supplement: Supplementary file 1 — ExCEL Trauma Survey Workshop Survey.docxTrauma Survey Demonstration.docxRole-Play Prebrief.docxNormal Trauma Survey.docxInjured Patient Trauma Survey.docx [file mep_2374-8265.11079-s001.zip › C. Role Play Prebrief.docx]

**Trauma Role Play Pre-brief**

Participants will perform primary and secondary trauma surveys, as per the 4 cases. Each member of the group should be responsible for performing one of the cases.

Facilitator Actions

Facilitator(s) should observe each step performed by the participant and provide feedback to the participant to ensure a complete and efficient assessment of each step. Sample scripting is provided. The facilitator should not volunteer information before the participant performs the physical exam that would elicit it.

Patient-Actor Actions

The patient-actor can decide which portions of the physical examination (s)he does not feel comfortable allowing the facilitators or participants to perform. For example, patient-actors can decline to allow palpation of femoral pulses, evaluation for pelvic mobility or palpation of chest wall or extremities. This should be discussed with the facilitators ahead of time and explained to the participants during the pre-brief.

**Facilitator should pre-brief the participants prior to starting the cases.**

Pre-Brief for Participants

- Each participant will perform one of the cases; remaining group members will observe and provide feedback or help to the team member running the case, as needed.
- Perform all aspects of the physical examination as you would on a patient. For scenarios involving patient-actors, sensitive areas of examination (e.g. assessment of the pelvis and chest) should be stated by the participant rather than performed directly.
- When patient-actors are involved, the patient-actor will provide physical exam findings (e.g. presence of ecchymosis on inspection or tenderness on palpation).
- When a manikin used, the facilitator will provide physical exam findings (e.g. presence of ecchymosis on inspection or tenderness on palpation).
- Information about the physical examination will only be provided when the patient-actor or facilitator observes appropriate evaluation. For example, information regarding tenderness will only be provided if the participant palpates the area.
- Performing procedures is not an objective of this skills station. Should a procedure be required, state the need for that intervention. The facilitator will state that the procedure was performed successfully and the case will then continue.
