# Supplementary material for: Excellence in Communication and Emergency Leadership (ExCEL): Pediatric Primary and Secondary Survey in Trauma Workshop for Residents
Source: MedEdPORTAL. 2021 Jan 22;17:11079. doi: 10.15766/mep_2374-8265.11079 (PMC7821439; doi:10.15766/mep_2374-8265.11079)
Supplement: Supplementary file 1 — ExCEL Trauma Survey Workshop Survey.docxTrauma Survey Demonstration.docxRole-Play Prebrief.docxNormal Trauma Survey.docxInjured Patient Trauma Survey.docx [file mep_2374-8265.11079-s001.zip › D. Normal Trauma Survey.docx]

**Normal Trauma Survey**

Objective: Participants should demonstrate ability to perform primary and secondary trauma surveys.

*EMS phone rings*: “This is Rescue 6 coming in with a patient involved in an MVC. 17-year-old was the restrained driver. Minimal damage to the car, no airbags deployed. Patient ambulated on scene. We’ll be there in 3 minutes.”

*If requested*: Patient is boarded and collared. (S)He is awake and alert. No obvious injuries.

*If requested*: Vitals: HR 88 RR 18 BP 116 / 82 SpO_2_ 99% RA

Primary and Secondary Surveys:

Vitals: HR 88 RR 18 BP 116 / 82 SpO_2_ 99% RA

1. **Primary survey**:

| **Assessment** | **Expected Participant Action and *Statements*** |
| --- | --- |
| - 1. Airway | Ask patient a question. Get a clear response.  *State, “Airway intact”* |
| - 1. Breathing | Listen to bilateral lung fields.  *State, “Bilateral breath sounds”* |
| - 1. Circulation | Feel femoral pulses. (If patient-actor, the participant need only state that (s)he will palpate femoral pulse.)  *State, “2+ femoral pulses”.*  Obtain heart rate and blood pressure from nurse/tech.  *State, “Heart rate and blood pressure within normal limits for age”* |
| - 1. Disability | Ask patient name, place and date.  *State, “Alert and oriented to person, place and time”*  Observe for spontaneous eye opening, verbal response and motor response.  *State, “GCS 15”* (If using infant manikin, observe that patient is alert. *State,* *“Patient alert on AVPU, this correlates to GCS of 15”*) |
| - 1. Exposure | Remove all clothing. (If patient-actor, the participant need only state that (s)he will remove all clothing.)  *State, “No obvious injuries when patient exposed”* |

1. **Secondary survey**: Observe, palpate and, when necessary, auscultate

| - 1. Head | Inspect and palpate scalp to assess for tenderness, wounds, hematomas, or step-offs.  *State, “Atraumatic without tenderness”* |
| --- | --- |
| - 1. Face | Inspect for wounds, swelling or ecchymosis and palpate for tenderness or step-offs  *State, “No trauma, no tenderness, no bruises “* |
| - 1. Eyes | Use light to evaluate pupil size and reactivity.  *State, “Pupils equal, round and reactive to light – 4mm to 2mm bilaterally”* |
| - 1. Ears | Use otoscope bilaterally to evaluate for hemotympanum or bleeding of external auditory canal.  *State, “No hemotympanum bilaterally”* |
| - 1. Nose | Use otoscope bilaterally to evaluate for septal hematoma, bleeding or swelling.  *State, “No deformities, epistaxis or nasal septal hematoma”* |
| - 1. Mouth | Use light to evaluate for wounds, dental trauma. Ask the patient to bite down and describe if teeth align normally.  *State, “No bleeding or malocclusion. Teeth intact.”* |
| - 1. Neck | Palpate anterior neck for edema, swelling, and position of the trachea  *State, “Trachea midline”.*  Hold C-spine in place while palpating each posterior vertebral spine.  *State, “No midline C-spine tenderness or step-offs”* |
| - 1. Chest | Inspect chest wall for deformity, ecchymosis or wounds. Observe chest rise during breathing for symmetry. Palpate anterior chest wall for tenderness or crepitus. (If patient-actor, the participant need only state that (s)he will perform these steps).  *State, “Symmetric chest rise, no clavicular deformity or tenderness, no crepitus, no tenderness to palpation”* |
| - 1. Abdomen | Inspect abdomen for deformity, ecchymosis or wounds. Palpate in all 4 quadrants for tenderness. (If patient-actor, the participant need only state that (s)he will perform these steps).  *State, “No bruising, ecchymosis, seatbelt sign; no tenderness”* |
| - 1. Pelvis | Inspect for ecchymosis, deformity, asymmetry, or wounds. Palpate the pelvis for tenderness, Assess for mobility by compressing iliac crests gently. Inspect urethral meatus for blood. (If patient-actor, the participant need only state that (s)he will perform these steps).  *State, “Pelvis stable, no blood at the meatus, no perineal bruising or lacerations”* |
| - 1. Back | Inspect for ecchymosis, deformity, asymmetry or wounds. Palpate along spine for tenderness or step-offs.  *State, “No bruising, nontender without step-offs. Good gluteal tone”* |
| - 1. Extremities | For each extremity: Inspect for ecchymosis, deformity, or wounds. Palpate for tenderness.  *State, “No bruising, lacerations, deformities; no tenderness to upper and lower extremities.”*  For each extremity: Assess strength and sensation.  *State, “Strength 5/5 in upper and lower extremities bilaterally; sensation 5/5 in upper and lower extremities bilaterally”*  For each extremity: Assess range of motion of all joints  *State, “Full range of motion in all extremities”* |

**Discussion Questions** (to be prompted by ExCEL facilitator):

- What trauma level does this patient meet? (institution-specific)

**Reference**:

1. Advanced trauma life support (ATLS®). *J Trauma Acute Care Surg*. 2013;74(5):1363-1366. doi:10.1097/TA.0b013e31828b82f5S
