# Supplementary material for: Excellence in Communication and Emergency Leadership (ExCEL): Pediatric Primary and Secondary Survey in Trauma Workshop for Residents
Source: MedEdPORTAL. 2021 Jan 22;17:11079. doi: 10.15766/mep_2374-8265.11079 (PMC7821439; doi:10.15766/mep_2374-8265.11079)
Supplement: Supplementary file 1 — ExCEL Trauma Survey Workshop Survey.docxTrauma Survey Demonstration.docxRole-Play Prebrief.docxNormal Trauma Survey.docxInjured Patient Trauma Survey.docx [file mep_2374-8265.11079-s001.zip › E. Injured Patient Trauma Survey.docx]

**Case 1**: Abdominal Trauma

Objectives:

- Identify abdominal trauma with tenderness
- Identify need for abdominal imaging to evaluate for possible intra-abdominal injury

*EMS phone rings*: “This is Rescue 3 bringing in a 5-year-old who fell off the trampoline. Unwitnessed, unknown loss of consciousness. Parents found him lying next to the trampoline.”

*If requested*: Patient is boarded and collared. Difficult to examine. No obvious injuries.

*If requested*: Vitals: In process now.

Primary and Secondary Surveys:

Vitals: HR 122 RR 30 BP 98 / 70 SpO_2_ 97% RA

1. **Primary survey**:

| **Assessment** | **Patient-Actor/Manikin Status** | **Expected Participant action and *Statements*** |
| --- | --- | --- |
| - 1. Airway | Crying | Observe the patient crying.  *State, “Airway intact”* |
| - 1. Breathing |  | Listen to bilateral lung fields.  *State, “Bilateral breath sounds”* |
| - 1. Circulation |  | Palpate for femoral pulses. (If patient-actor, the participant need only state that (s)he will palpate femoral pulse.)  *State, “2+ femoral pulses”.*  Obtain heart rate and blood pressure from nurse/tech (to be provided by facilitator).  *State, “Heart rate and blood pressure within normal limits for age”* |
| - 1. Disability | Alert, crying for mom | Observe the patient crying; ask patient name and place.  *State, “Alert and oriented to person, place and time”*  Observe for spontaneous eye opening, verbal response and motor response.  *State, “Alert on AVPU, correlates to GCS 15”* |
| - 1. Exposure | Bruise to mid-abdomen | Remove all clothing. (If patient-actor, the participant need only state that (s)he will remove clothing). Observe ecchymosis to abdomen.  *State, “Bruise to mid-abdomen”* |

1. **Secondary survey**: Observe, palpate and, when necessary, auscultate

| - 1. Head |  | Inspect and palpate scalp to assess for tenderness, wounds, hematomas, or step-offs.  *State, “Atraumatic without tenderness”* |
| --- | --- | --- |
| - 1. Face |  | Inspect for wounds, swelling or ecchymosis and palpate for tenderness or step-offs  *State, “No trauma, no tenderness, no bruises “* |
| - 1. Eyes |  | Use light to evaluate pupil size and reactivity.  *State, “Pupils equal, round and reactive to light – 4mm to 2mm bilaterally”* |
| - 1. Ears |  | Use otoscope bilaterally to evaluate for hemotympanum or bleeding of external auditory canal.  *State, “No hemotympanum bilaterally”* |
| - 1. Nose |  | Use otoscope bilaterally to evaluate for septal hematoma, bleeding or swelling.  *State, “No deformities, epistaxis or nasal septal hematoma”* |
| - 1. Mouth |  | Use light to evaluate for wounds, dental trauma. Ask the patient to bite down and describe if teeth align normally.  *State, “No bleeding or malocclusion. Teeth intact.”* |
| - 1. Neck |  | Palpate anterior neck for edema, swelling, and position of the trachea  *State, “Trachea midline”.*  Hold C-spine in place while palpating each posterior vertebral spine.  *State, “No midline C-spine tenderness or step-offs”* |
| - 1. Chest |  | Inspect chest wall for deformity, ecchymosis or wounds. Observe chest rise during breathing for symmetry. Palpate anterior chest wall for tenderness or crepitus. (If patient-actor, the participant need only state that (s)he will perform these steps).  *State, “Symmetric chest rise, no clavicular deformity or tenderness, no crepitus, no tenderness to palpation”* |
| - 1. Abdomen | Describe ecchymosis to abdomen and tenderness to palpation | Inspect abdomen for deformity, ecchymosis or wounds; **observe bruising to mid-abdomen**. Palpate in all 4 quadrants for tenderness, **recognize diffuse tenderness throughout abdomen**. (If patient-actor, the participant need only state that (s)he will perform these steps).  *State, “abdominal bruising, diffuse abdominal tenderness”* |
| - 1. Pelvis |  | Inspect for ecchymosis, deformity, asymmetry, or wounds. Palpate the pelvis for tenderness, Assess for mobility by compressing iliac crests gently. Inspect urethral meatus for blood. (If patient-actor, the participant need only state that (s)he will perform these steps).  *State, “Pelvis stable, no blood at the meatus, no perineal bruising or lacerations”* |
| - 1. Back |  | Inspect for ecchymosis, deformity, asymmetry or wounds. Palpate along spine for tenderness or step-offs.  *State, “No bruising, nontender without step-offs. Good gluteal tone”* |
| - 1. Extremities |  | For each extremity: Inspect for ecchymosis, deformity, or wounds. Palpate for tenderness.  *State, “No bruising, lacerations, deformities; no tenderness to upper and lower extremities.”*  For each extremity: Assess strength and sensation.  *State, “Strength 5/5 in upper and lower extremities bilaterally; sensation 5/5 in upper and lower extremities bilaterally”*  For each extremity: Assess range of motion of all joints  *State, “Full range of motion in all extremities”* |

Discussion Questions (to be prompted by ExCEL facilitator):

- What trauma level does this patient meet? (institution-specific)
- What imaging modalities are options for this hemodynamically stable patient with evidence of abdominal trauma?

**Case 2:** Possible C-Spine Injury

Objectives:

- Identify possible C-spine injury
- Identify need for C-spine imaging due to C-spine tenderness and intoxication

*EMS phone rings*: “This is Rescue 2 coming in with an 18-year-old intoxicated patient. (S)He was at a party and fell down the stairs.”

*If requested*: Patient boarded and collared. Intoxicated and difficult to examine. No obvious injuries.

*If requested*: Vitals: 108 RR 14 BP 128 / 86 SpO_2_ 99% RA

Primary and Secondary Surveys:

Vitals: HR 108 RR 14 BP 128 / 86 SpO_2_ 99% RA

1. **Primary survey**:

| **Assessment** | **Patient-Actor/Manikin Status** | **Expected Participant action and *Statements*** |
| --- | --- | --- |
| - 1. Airway | Intoxicated, slurring speech | Ask patient a question. Get a verbal response with some **slurring**.  *State, “Airway intact”* |
| - 1. Breathing |  | Listen to bilateral lung fields.  *State, “Bilateral breath sounds”* |
| - 1. Circulation |  | Palpate for femoral pulses. (If patient-actor, the participant need only state that (s)he will palpate femoral pulse.)  *State, “2+ femoral pulses”.*  Obtain heart rate and blood pressure from nurse/tech (to be provided by facilitator).  *State, “Heart rate and blood pressure within normal limits for age”* |
| - 1. Disability | Combative, uncooperative, intoxicated | Observe patient is **combative and uncooperative, consistent with intoxication.**  *State, “Uncooperative. Not oriented to person, place and time”*  Observe for spontaneous eye opening, verbal response and motor response.  *State, “GCS 14 for confused conversation”* |
| - 1. Exposure |  | Remove all clothing. (If patient-actor, the participant need only state that (s)he will remove clothing). Observe ecchymosis to abdomen.  *State, “Bruise to mid-abdomen”* |

1. **Secondary survey**: Observe, palpate and, when necessary, auscultate

| - 1. Head |  | Inspect and palpate scalp to assess for tenderness, wounds, hematomas, or step-offs.  *State, “Atraumatic without tenderness”* |
| --- | --- | --- |
| - 1. Face | Describe bruise on right cheek | Inspect for wounds, swelling or ecchymosis and palpate for tenderness or step-offs. Observe **bruise to right cheek**  *State, “Bruise to R cheek, tenderness to palpation“* |
| - 1. Eyes |  | Use light to evaluate pupil size and reactivity.  *State, “Pupils equal, round and reactive to light – 4mm to 2mm bilaterally”* |
| - 1. Ears |  | Use otoscope bilaterally to evaluate for hemotympanum or bleeding of external auditory canal.  *State, “No hemotympanum bilaterally”* |
| - 1. Nose |  | Use otoscope bilaterally to evaluate for septal hematoma, bleeding or swelling.  *State, “No deformities, epistaxis or nasal septal hematoma”* |
| - 1. Mouth |  | Use light to evaluate for wounds, dental trauma. Ask the patient to bite down and describe if teeth align normally.  *State, “No bleeding or malocclusion. Teeth intact.”* |
| - 1. Neck | React with pain upon palpation of C-spine | Palpate anterior neck for edema, swelling, and position of the trachea  *State, “Trachea midline”.*  Hold C-spine in place while palpating each posterior vertebral spine and note **midline tenderness at C3-6**.  *State, “C spine tenderness to palpation at C3-6”* |
| - 1. Chest |  | Inspect chest wall for deformity, ecchymosis or wounds. Observe chest rise during breathing for symmetry. Palpate anterior chest wall for tenderness or crepitus. (If patient-actor, the participant need only state that (s)he will perform these steps).  *State, “Symmetric chest rise, no clavicular deformity or tenderness, no crepitus, no tenderness to palpation”* |
| - 1. Abdomen |  | Inspect abdomen for deformity, ecchymosis or wounds. Palpate in all 4 quadrants for tenderness. (If patient-actor, the participant need only state that (s)he will perform these steps).  *State, “No bruising, ecchymosis, seatbelt sign; no tenderness”* |
| - 1. Pelvis |  | Inspect for ecchymosis, deformity, asymmetry, or wounds. Palpate the pelvis for tenderness, Assess for mobility by compressing iliac crests gently. Inspect urethral meatus for blood. (If patient-actor, the participant need only state that (s)he will perform these steps).  *State, “Pelvis stable, no blood at the meatus, no perineal bruising or lacerations”* |
| - 1. Back |  | Inspect for ecchymosis, deformity, asymmetry or wounds. Palpate along spine for tenderness or step-offs.  *State, “No bruising, nontender without step-offs. Good gluteal tone”* |
| - 1. Extremities |  | For each extremity: Inspect for ecchymosis, deformity, or wounds. Palpate for tenderness.  *State, “No bruising, lacerations, deformities; no tenderness to upper and lower extremities.”*  For each extremity: Assess strength and sensation.  *State, “Strength 5/5 in upper and lower extremities bilaterally; sensation 5/5 in upper and lower extremities bilaterally”*  For each extremity: Assess range of motion of all joints  *State, “Full range of motion in all extremities”* |

Discussion Questions (to be prompted by ExCEL facilitator):

- What trauma level does this patient meet? (institution specific)
- What imaging modalities are options for this hemodynamically stable patient with altered mental status and evidence of C-spine injury?

**Case 3**: Traumatic Pneumothorax

Objectives:

- Recognize shock in trauma patient
- Identify tension pneumothorax
- Understand need for needle decompression immediately (prior to completion of survey)

*EMS phone rings*: “This is Rescue 4 coming in with a 17-year-old restrained driver in an MVC - car vs pole.  Extensive damage to the car, + airbags deployed. Patient was moaning on scene, non-ambulatory. We’ll be there in 3 minutes.”

*If requested*: Patient is boarded and collared.  (S)He is not opening his eyes or moving spontaneously, mumbling incoherently.

*If requested*: Vitals: HR 135  RR 25 BP 80 /42 SpO_2_ 86% RA

Primary and Secondary Surveys:

Vitals: 135  RR 25 BP 80 /42 SpO_2_ 86% RA

1. **Primary survey**:

| **Assessment** | **Patient-Actor/Manikin Status** | **Expected Participant action and *Statements*** |
| --- | --- | --- |
| - 1. Airway | Unresponsive | Ask patient a question. No response. Examine airway, note no obstruction.  *State, “Airway patent”* |
| - 1. Breathing | Describe trachea deviated to left, splinting with breathing, no breath sounds on the right | Listen to bilateral lung fields and **note absence of breath sounds on the right and tracheal deviation to the left.**  *State, “absent breath sounds on the right”* |

**Expected Participant Actions: STOP Primary Survey**

- State that needle decompression is required
- Call pediatric trauma team
- Facilitator explains that appropriate needle decompression performed
- State need for chest tube and that trauma surgery is preparing for that procedure

Lifesaver: if the participants do not stop their primary survey to perform needle decompression, the facilitator will stop the case for feedback and discussion.

Repeat vitals HR 110, BP 98/58, RR 22, O2 95%

Continue with **primary survey**

| - 1. Circulation |  | Feel femoral pulses. (If patient-actor, the participant need only state that (s)he will palpate femoral pulse.)  *State, “2+ femoral pulses”.*  Obtain heart rate and blood pressure from nurse/tech.  *State, “Heart rate and blood pressure within normal limits for age”* |
| --- | --- | --- |
| - 1. Disability | Opening eyes spontaneously, confused but able to answer questions, obeys commands to move extremities | Ask patient name, place and date.  *State, “Alert and oriented to person, place and time”*  Observe for spontaneous eye opening, verbal response and motor response.  *State, “GCS 14”* |
| - 1. Exposure | Describe bruise to right chest wall, needle right chest, midclavicular line | Remove all clothing. (If patient-actor, the participant need only state that (s)he will remove all clothing.) Observe **bruise to R chest** and **needle from needle decompression**.  *State, “Bruise to right chest wall and needle from needle decompression”* |

1. **Secondary survey**: Observe, palpate and, when necessary, auscultate

| - 1. Head |  | Inspect and palpate scalp to assess for tenderness, wounds, hematomas, or step-offs.  *State, “Atraumatic without tenderness”* |
| --- | --- | --- |
| - 1. Face |  | Inspect for wounds, swelling or ecchymosis and palpate for tenderness or step-offs  *State, “No trauma, no tenderness, no bruises “* |
| - 1. Eyes |  | Use light to evaluate pupil size and reactivity.  *State, “Pupils equal, round and reactive to light – 4mm to 2mm bilaterally”* |
| - 1. Ears |  | Use otoscope bilaterally to evaluate for hemotympanum or bleeding of external auditory canal.  *State, “No hemotympanum bilaterally”* |
| - 1. Nose |  | Use otoscope bilaterally to evaluate for septal hematoma, bleeding to swelling.  *State, “No deformities, epistaxis or nasal septal hematoma”* |
| - 1. Mouth |  | Use light to evaluate for wounds, dental trauma. Ask the patient to bite down and describe if teeth align normally.  *State, “No bleeding or malocclusion. Teeth intact.”* |
| - 1. Neck |  | Palpate anterior neck for edema, swelling, and position of the trachea. **Trachea no longer deviated.**  *State, “Trachea midline”.*  Hold C-spine in place while palpating each posterior vertebral spine.  *State, “No midline C-spine tenderness or step-offs”* |
| - 1. Chest | Needle located in right anterior chest wall | Inspect chest wall for deformity, ecchymosis or wounds. Observe **needle in anterior chest wall**, chest rise during breathing for symmetry. Palpate anterior chest wall for tenderness or crepitus. (If patient-actor, the participant need only state that (s)he will perform these steps).  *State, “Symmetric chest rise, no clavicular deformity or tenderness, no crepitus, no tenderness to palpation. ”* |
| - 1. Abdomen |  | Inspect abdomen for deformity, ecchymosis or wounds. Palpate in all 4 quadrants for tenderness. (If patient-actor, the participant need only state that (s)he will perform these steps).  *State, “No bruising, ecchymosis, seatbelt sign; no tenderness”* |
| - 1. Pelvis |  | Inspect for ecchymosis, deformity, asymmetry, or wounds. Palpate the pelvis for tenderness, Assess for mobility by compressing iliac crests gently. Inspect urethral meatus for blood. (If patient-actor, the participant need only state that (s)he will perform these steps).  *State, “Pelvis stable, no blood at the meatus, no perineal bruising or lacerations”* |
| - 1. Back |  | Inspect for ecchymosis, deformity, asymmetry or wounds. Palpate along spine for tenderness or step-offs.  *State, “No bruising, nontender without step-offs. Good gluteal tone”* |
| - 1. Extremities |  | For each extremity: Inspect for ecchymosis, deformity, or wounds. Palpate for tenderness.  *State, “No bruising, lacerations, deformities; no tenderness to upper and lower extremities.”*  For each extremity: Assess strength and sensation.  *State, “Strength 5/5 in upper and lower extremities bilaterally; sensation 5/5 in upper and lower extremities bilaterally”*  For each extremity: Assess range of motion of all joints  *State, “Full range of motion in all extremities”* |

Discussion Questions (to be prompted by ExCEL facilitator):

- What trauma level does this patient meet? (institution specific)
- What procedure must be performed in this hemodynamically unstable patient with absent breath sounds on one side?

**Reference**:

1. Advanced trauma life support (ATLS®). *J Trauma Acute Care Surg*. 2013;74(5):1363-1366. doi:10.1097/TA.0b013e31828b82f5S
